# Conversion of oat (*Avena sativa* L.) haploid embryos into plants in relation to embryo developmental stage and regeneration media

**DOI:** 10.1007/s11627-016-9788-z

**Published:** 2016-11-04

**Authors:** Angelika Noga, Edyta Skrzypek, Marzena Warchoł, Ilona Czyczyło-Mysza, Kinga Dziurka, Izabela Marcińska, Katarzyna Juzoń, Tomasz Warzecha, Agnieszka Sutkowska, Zygmunt Nita, Krystyna Werwińska

**Affiliations:** 1 Department of Biotechnology, Polish Academy of Sciences, The Franciszek Górski Institute of Plant Physiology, Niezapominajek 21, 30-239 Kraków, Poland; 2 Department of Plant Breeding and Seed Science, University of Agriculture, Łobzowska 24, 31-140 Kraków, Poland; 3 Plant Breeding Strzelce Ltd., PBAI Group, Główna 20, 99-307 Strzelce, Poland

**Keywords:** *Avena sativa* L., DH lines, Haploid embryos, Growth regulators

## Abstract

Obtaining oat DH lines is only effective via wide crossing with maize. Seven hundred haploid embryos from 21 single F_1_ progeny obtained from wide crosses with maize were isolated, divided into four groups according to their size (<0.5 mm, 0.5–0.9 mm, 1.0–1.4 mm, and ≥1.5 mm), and transferred into 190–2 regeneration medium with different growth regulators: 0.5 mg L^−1^ kinetin (KIN) and 0.5 mg L^−1^ 1-naphthaleneacetic acid (NAA); 1 mg L^−1^ zeatin (ZEA) and 0.5 mg L^−1^ NAA; or 1 mg L^−1^ dicamba (DIC), 1 mg L^−1^ picloram (PIC), and 0.5 mg L^−1^ kinetin (KIN). Among all isolated embryos, approximately 46.1% were between 1.0–1.4 mm, while the smallest group of embryos (7.1%) were those <0.5 mm. The ability of haploid embryos to germinate varied depending on oat genotypes and the size of embryos. Haploid embryos <0.5 mm were globular and did not germinate, whereas embryos ≥1.5 mm had clearly visible coleoptiles, radicles, and scutella, and were able to germinate. Germination of oat haploid embryos varied depending on growth regulators in the regeneration medium. Most haploid embryos germinated on medium with 0.5 mg L^−1^ NAA and 0.5 mg L^−1^ KIN, while the fewest germinated on medium with 1 mg L^−1^ DIC, 1 mg L^−1^ PIC, and 0.5 mg L^−1^ KIN. One hundred thirty germinated haploid embryos converted into haploid plants. Fifty oat DH lines were obtained after colchicine treatment.

**Electronic supplementary material:**

The online version of this article (doi:10.1007/s11627-016-9788-z) contains supplementary material, which is available to authorized users.

## Introduction

Oat (*Avena sativa* L.) is one of the major cereal crops in the world and belongs to the Poaceae family. It is ranked sixth after wheat (*Triticum aestivum* L.), maize (*Zea mays* L.), rice (*Oryza sativa* L.), barley (*Hordeum vulgare* L.), and sorghum (*Sorghum bicolor* Moench). Oat can be used not only as a valuable forage for animals but also for human consumption as a source of valuable nutrients (Boczkowska and Traczyk 2013). Oat has a beneficial effect on human health due to its protein, carbohydrate, fiber, vitamin, and mineral content (Peterson 2004). During the last 50 yr, global production of oats has decreased by 50% (Boczkowska and Traczyk 2013). The wide range of oat applications results in a great demand for new cultivars. Haploidization is one of the methods of creating new cultivars that allows decreasing the production time of new cultivars by as much as 5–8 vegetative seasons. Currently, doubled haploid (DH) technology for practical breeding is routinely available in rapeseed (*Brassica napus* L.), barley, and wheat (Forster and Thomas 2005). However, oat, as well as rye (*Secale cereale* L.), and triticale (× *Triticosecale*Wittm. ex *A. Camus*) are examples of crops where DH technologies are less advanced, but still hundreds of DHs can be obtained (Tuvesson *et al.* 2007). Wide crossing with maize is the main and most-effective method for producing oat DH lines (Rines 2003; Marcińska *et al.* 2013; Nowakowska *et al.* 2015; Warchoł *et al.* 2016; Skrzypek *et al.* 2016).

Although the wide crossing method has many advantages, certain challenges also exist. There are several barriers that operate at pre- and post-fertilization levels that make wide crossing difficult (Chawla 2002). The pre-fertilization barriers include all factors that hinder effective fertilization, such as the inhibition of pollen tube growth by the stigma or upper style, while the post-fertilization barriers hinder or retard the development of the zygote and normal development of the seed. Embryo abortion occurs quite frequently as a result of unsuccessful crosses in breeding (Taji *et al.* 2001). Although most of these cases undergo successful fertilization and early embryo development occurs, a number of subsequent irregular events can lead to the eventual death of the embryo and as a consequence collapse of the seed. A major cause of early embryo abortion is a failure of normal endosperm development.

Embryos have been recovered from interspecific hybrids in many species, and success has been achieved also with intergeneric hybrids of barley and rye, and of wheat and rye (Taji *et al.* 2001). As the endosperm usually fails to develop in seeds, embryos must be transferred to an artificial culture medium to allow them to grow under optimum culture conditions (Sidhu 2006). This procedure is referred to as embryo rescue. The most important aspect of the embryo rescue technique is the selection of regeneration medium necessary to sustain the continued growth of the embryo (Chawla 2002). Media can be supplemented with vitamins, growth regulators, and with natural extracts to ensure optimum conditions for embryo development (Bridgen 1994). Carbohydrates serve as the carbon source in the culture media, among which sucrose is the most common. Other sugars can be used instead of or in addition to sucrose (Reed 2005). Sugars are used in a concentration range of 2–12%. Generally, the younger the embryo, the higher the medium osmolarity is required. Agar is the most commonly used media-solidifying agent, in concentrations of 0.5–1.5%. The concentration of agar may affect embryo growth. High concentrations may inhibit growth of haploid embryos due to reduced water access, quality of agar, or contaminating salts (Bridgen 1994). Among the growth regulators, the concentrations of auxins and cytokinins, which control cell division and morphogenesis, are particularly important *in vitro*. Among auxins, indoleacetic acid (IAA) is the most widely used. This natural auxin can be replaced by synthetic auxin analogs, such as 2,4-dichlorophenoxyacetic acid (2,4-D), 1-naphaleneacetic acid (NAA), 3,6-dichloro-2-methoxybenzoic acid (DIC), 4-amino-3,5,6-trichloro-2-pyridinecarboxylic acid (PIC) or their combinations. Kinetin (KIN), 6-benzylaminopurine (BAP), and zeatin (ZEA) are commonly used cytokinins (Żur *et al.* 2015).

According to Rines (2003), the need to overcome the post-fertilization barriers and to select the appropriate regeneration medium for embryo development makes the germination period a critical stage in the development of haploid embryos of oat. What is more, the development of haploid embryos is not synchronized despite the same time of pollination with maize and isolation into regeneration medium. The percentage of embryo recovery usually ranges between 2 and 10% of maize-pollinated oat florets. The rate of germination of these embryos into vigorous plants is typically low and falls below 20%. The present research was conducted to increase the efficiency of oat haploid embryo conversion into haploid plants. The aim of this study was to analyze the possible correlations between the type of regeneration medium and germination capacity of oat haploid embryos in various developmental stages.

## Material and Methods

The experiments were performed on single F_1_ oat progeny obtained from 21 separate crosses: STH 4.8456/1, STH 4.8456/2, STH 4.8457/1, STH 4.8457/2, STH 5.8421, STH 5.8422, STH 5.8423, STH 5.8424, STH 5.8425, STH 5.8426, STH 5.8427, STH 5.8428, STH 5.8429, STH 5.8430, STH 5.8432, STH 5.8436, STH 5.8440, STH 5.8449, STH 5.8450, STH 5.8458, and STH 5.8460. All were derived from Plant Breeding Strzelce Ltd., PBAI Group, Strzelce, Łódź Voivodeship, Poland. Five seeds of each single F_1_ oat progeny were sown singly into a mixture of soil with sand (3:1 *v/v*) in 3-L pots. Oat plants were grown in controlled conditions in a greenhouse at temperatures of 21/17°C (day/night), and maize cultivar Waza plants were grown in controlled conditions in a greenhouse at temperatures of 25–28/17°C (day/night). Plants were grown under natural (solar) light during the day and under sodium lamps (400 W, Philips SON-T AGRO, Philips Lighting, Eidhoven, the Netherlands) to maintain a 16-h photoperiod on cloudy days. The light intensity was 800 μmol m^−2^ s^−1^. All plants were fertilized with Hoagland liquid medium (Hoagland and Arnon, 1938) once per week.

Oat haploid plants were obtained using the wide crossing method by pollination with maize, as described by Marcińska *et al.* (2013). Three weeks after floret pollination, enlarged ovaries were collected, surface-sterilized in 70% (*v/v*) ethanol (1 min), then in a 2.5% (*w/v*) solution of calcium hypochlorite (65% Ca(OCl)2 commercial product, Sigma-Aldrich®, Darmstadt, Germany) (8 min), and subsequently washed three times with sterile water. Then, haploid embryos were isolated, divided into four groups according to their size (<0.5 mm, 0.5–0.9 mm, 1.0–1.4 mm, and ≥1.5 mm), and transferred to 60 × 15 mm Petri dishes containing liquid 190–2 media (Zhuang and Xu, 1983) with 9% (*w/v*) maltose, 0.6% (*w/v*) agar and different growth regulators. The first medium contained 0.5 mg L^−1^ KIN and 0.5 mg L^−1^ NAA; the second medium contained 1 mg L^−1^ ZEA and 0.5 mg L^−1^ NAA; and the third medium contained 1 mg L^−1^ DIC, 1 mg L^−1^ PIC, and 0.5 mg L^−1^ KIN. The pH of all media was adjusted to 6.0 before autoclaving at 135°C at 360 kPa for 4.5 min (Microjet, Enbio Technology Ltd., Gdańsk, Poland). The growth regulators and vitamins were filtered into the media, which were cooled after autoclaving, with sterile syringe filters of 0.2-μm mesh (Whatman® Puradisc 30 syringe filters, Sigma-Aldrich®, Darmstadt, Germany). Approximately, equal proportions of haploid embryos of each size for each genotype were transferred to the three media. Haploid embryos were germinated in an *in vitro* chamber (21°C, with a 16-h photoperiod and light intensity of 100 μmol m^2^ s^−1^). The germination capacity of haploid embryos was evaluated for each type of medium. The observation of haploid embryos was carried out under a stereomicroscope (SMZ 1500, Nikon, Tokyo, Japan) and photographs were taken using a digital CCD camera (DS-Ri1, Nikon, Tokyo, Japan).

The plants developed from haploid embryos were moved to MS medium (Murashige and Skoog, 1962) with 0.6% (*w/v*) agar. Subsequently, the plants were acclimated to natural conditions by transferring them to wet perlite (Zakłady Górniczo-Metalowe ZĘBIEC S.A., Zębiec, Poland) and next to universal soil (Ziemia uniwersalna, Ekoziem, Jurków, Poland). After acclimation, roots of haploid plants were dipped in colchicine for chromosome doubling. They were treated for 7.5 h with a 500 ml aqueous solution of 0.1% (*w/v*) colchicine, 40 mL L^−1^ dimethyl sulfoxide (DMSO), a 80 μl L^−1^ of Tween, and 0.025 g L^−1^ gibberellic acid. Then, the plant roots were washed in running water for 48 h. Colchicine treatment was performed at 25°C and light intensity of 80–100 μmol m^2^ s^−1^. After the root washing, the plants were grown in the greenhouse until maturation.

The ploidy level of plants was evaluated using a MACSQuant flow cytometer (MACSQuant, Miltenyi Biotec GmbH, Bergisch Gladbach, Germany). For each plant, approximately 10–15 mg of young leaves was placed in a 60-mm glass Petri dish. Lysis buffer (45 mM MgCl_2_, 30 mM sodium citrate, 20 mM 4-morpholinepropane sulfonic acid, 0.1% (*v/v*) Triton X-100 in distilled water, pH 7.0) (Galbraith *et al.* 1983) was added to the plant material. The tissue was chopped with a razor blade and then filtered into 5-mL tubes with a pre-separation filter composed of 30-μm nylon mesh (Miltenyi Biotec GmbH, Bergisch Gladbach, Germany). The nuclei suspension (1.0 mL) was stained with 30 μL of a 2% (*w/v*) aqueous propidium iodide (PI) solution. Aliquots (50 μL) of stained nuclei were gently shaken and ploidy was analyzed using a flow cytometer (MACSQuant, Miltenyi Biotec GmbH, Bergisch Gladbach, Germany) equipped with an air-cooled laser (488 nm, fluorescence channel 4). In total, the fluorescence of at least 10,000 nuclei was analyzed in each sample. The control sample, a plant known to be diploid, was used to set the diploid gate. Plants with doubled chromosomes after colchicine treatment and that produced seeds were defined as DH lines.

All reagents used in the experiment were obtained from Sigma-Aldrich®.

Analysis of variance was carried out for germination of haploid embryos in relation to oat genotype, type of regeneration medium, and size of embryos. The size of embryos was also analyzed depending on the genotype. The results were analyzed using the Duncan test at *p* ≤ 0.05 using the STATISTICA 12.0 software package (Stat-Soft, Inc., Tulsa, OK).

## Results

The analysis of variance showed that the size of haploid embryos depended significantly on the genotype. The germination capacity of haploid embryos strongly varied between the oat genotypes (*p* ≤ 0.01) and size of embryos (*p* ≤ 0.001). The type of regeneration medium did not significantly affect haploid embryo germination. There was no interaction between the type of regeneration medium and size of embryos on the germination capacity (data not shown).

Seven hundred and twenty oat panicles (17,904 florets) from 21 oat genotypes were emasculated and pollinated with maize pollen (Table S1). A total of 700 haploid embryos were obtained from all genotypes, of which 133 germinated. The number of haploid embryos obtained from each genotype ranged from 5 to 83 and average embryo formation per genotype was 33.33 (Table 1). An average of 6.33 embryos per genotype germinated and half were acclimated to natural conditions. The highest number of germinated haploid embryos was observed for the STH 5.8425 and STH 5.8429 genotypes (21 and 22, respectively), whereas germination was not observed only for the STH 5.8450 genotype (Table S1). One hundred and thirty germinated haploid embryos survived the conversion into plants. All regenerated plants were green. Sixty-eight haploid plants survived the process of acclimatization to natural conditions and transplanting to perlite, and 59 survived the successive transplant to the soil.

**Table 1.** The numbers and percentages of haploid embryos, plants, and DH lines at various developmental stages

Trait	Mean		Minimum		Maximum	
	*n*	Percentage per floret	*n*	Percentage per floret	*n*	Percentage per floret
Haploid embryos	33.33	3.82	5	1.03	83	6.54
Germinated haploid embryos	6.33	0.67	0	0	22	1.76
Haploid plants on MS0	6.19	0.66	0	0	21	1.64
Haploid plants in perlite	3.24	0.33	0	0	10	0.80
Haploid plants in soil	2.81	0.29	0	0	9	0.70
DH plants after colchicine treatment	2.38	0.24	0	0	6	0.58
DH lines	2.14	0.22	0	0	6	0.58

The average percentage of haploid embryos formation per emasculated floret was 3.82 and ranged from 1.03–6.54% (Table S2). The percentage of germinated embryos per emasculated floret was significantly lower (0.67%) and varied between genotypes from 0 to 1.76%. Almost all germinated embryos developed into haploid plants. The percentage of haploid plants per emasculated floret (0.66%) was similar to the percentage of germinated embryos per emasculated floret (0.67%) (Table 1). Almost half of the haploid plants survived the process of acclimatization to natural conditions. Transplanting to wet perlite was the most critical step of acclimation. Plants of two genotypes, STH 5.8422 and STH 5.8460, did not survive the acclimatization (Table S2). Plants from two other genotypes, STH 5.8456/1 and STH 5.8428, died as a result of colchicine treatment.

**Figure 1.**
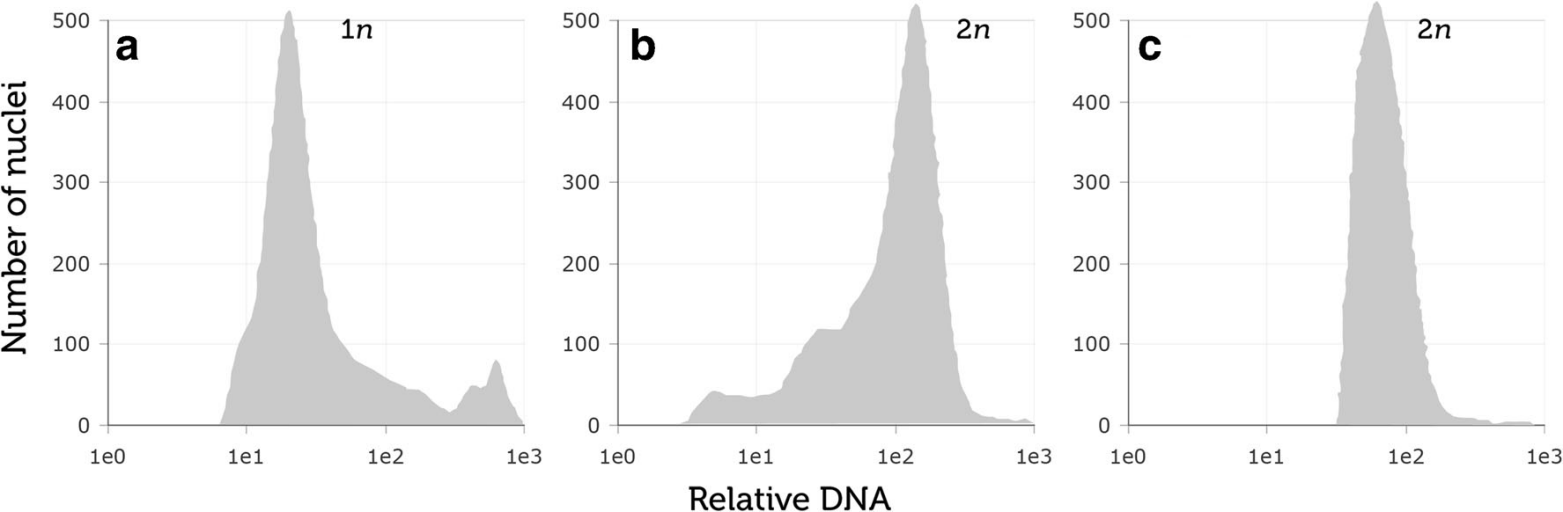
Flow cytometry histograms of oat plants: (*a*) haploid 1*n*; (*b*) doubled haploid 2*n*; and (*c*) control 2*n*.

The ploidy of the plants before (Fig. 1 *a*) and after colchicine treatments (Fig. 1 *b*) was compared with control diploid oat plants (Fig. 1 *c*). Cytometrical analysis confirmed that the colchicine treatment doubled chromosomes of all tested plants. Forty-five plants survived chromosome doubling by colchicine, and all were DH lines. DH lines were not obtained from five genotypes: STH 5.8422, STH 5.8424, STH 5.8428, STH 5.8450, and STH 5.8460 (Table S1). The average efficiency of DH line production was 0.22% and ranged from 0.11% for the STH 5.8457/1 genotype to 0.58% for the STH 4.8456/2 genotype. Plants from all genotypes developed seeds, with the exception of STH 5.8424.

**Figure 2.**
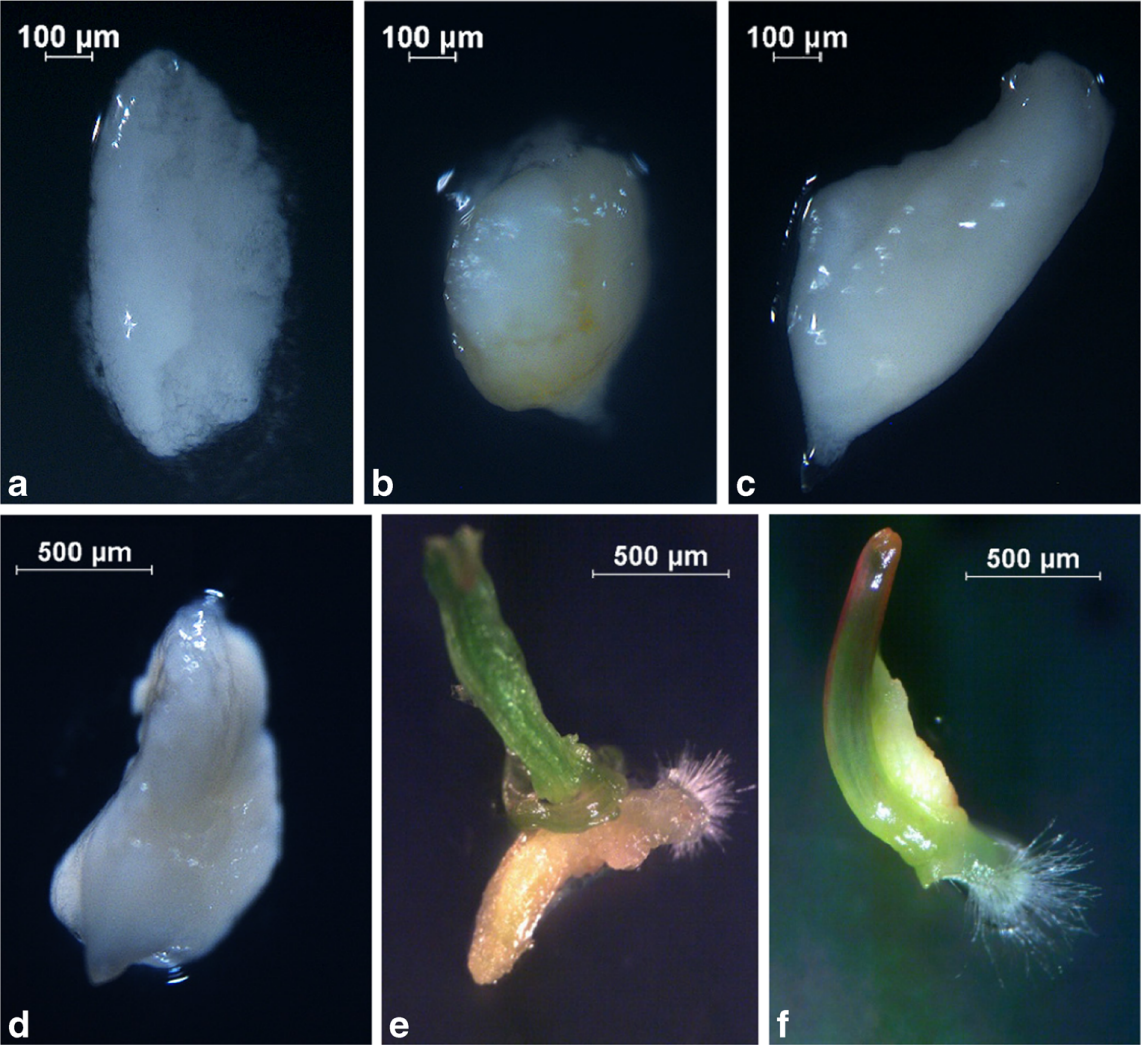
Oat × maize wide crossing for DH oat production: (*a*) endosperm without haploid embryo; (*b*) globular haploid embryo; (*c*) haploid embryo without visible coleoptile and radicle; (*d*) haploid embryo with coleoptile, radicle, and scutellum; and (*e*, *f*) germinated haploid embryos.

Although haploid embryo isolation was carried out at the same time (3 wk after floret pollination), the embryos did not develop equally in all genotypes and varied in size. All haploid seeds lacked endosperm. In some cases, only traces of endosperm were observed without the embryo (Fig. 2 *a*). Embryos smaller than 0.5 mm were globular (Fig. 2 *b*); those from 0.5–1.4 mm were longitudinal and without visible coleoptiles and radicles (Fig. 2 *c*); and those bigger than 1.5 mm were longitudinal with visible coleoptiles, radicles, and scutella (Fig. 2 *d*). Embryos were defined as converted into plants based on the apical meristem development (Fig. 2 *e*, *f*). The smallest group (50 of 700; 0.7%) consisted of embryos <0.5 mm (Table 2). There were 158 haploid embryos in the 0.5–0.9 mm group (22.6% of all haploid embryos). The largest group, nearly half of all haploid embryos (323; 46.1%), was 1.0–1.4 mm. These embryos were formed by the majority of oat genotypes. One hundred and sixty-nine haploid embryos were ≥1.5 mm, which amounted to 24.1% of all embryos. The developmental stage of haploid embryos depended on the genotype. The highest percentage of haploid embryos <0.5 mm and between 0.5 and 0.9 mm was observed from STH 5.8456/2, STH 5.8450, and STH 5.8458 genotypes. The largest number of fully developed haploid embryos in the 1.0–1.4 mm and ≥1.5 mm groups was recorded for STH 5.8424, STH 5.8425, STH 5.8426, STH 5.8427, and STH 5.8429.

**Table 2.** The numbers and percentages of oat haploid embryos according to their size and genotype

Genotype	Number of haploid embryos according to size [mm]					Percentage of haploid embryos according to size [mm]			
	< 0.5	0.5–0.9	1.0–1.4	≥1.5	∑	<0.5	0.5–0.9	1.0–1.4	≥1.5
STH 4.8456/1	2	8	18	1	29	6.9	27.6	62.1	3.4
STH 4.8456/2	3	14	5	6	28	10.7	50.0	17.9	21.4
STH 4.8457/1	1	6	14	9	30	3.3	20.0	46.7	30.0
STH 4.8457/2	0	15	25	7	47	0.0	31.9	53.2	14.9
STH 5.8421	4	7	12	9	32	12.5	21.9	37.5	28.1
STH 5.8422	2	0	3	1	6	33.3	0.0	50.0	16.7
STH 5.8423	7	12	24	4	47	14.9	25.5	51.1	8.5
STH 5.8424	1	10	18	15	44	2.3	22.7	40.9	34.1
STH 5.8425	1	8	54	21	84	1.2	9.5	64.3	25.0
STH 5.8426	2	7	14	10	33	6.1	21.2	42.4	30.3
STH 5.8427	0	4	20	22	46	0.0	8.7	43.5	47.8
STH 5.8428	1	5	7	0	13	7.7	38.5	53.8	0.0
STH 5.8429	1	9	26	28	64	1.6	14.1	40.6	43.8
STH 5.8430	1	3	8	5	17	5.9	17.6	47.1	29.4
STH 5.8432	4	7	11	7	29	13.8	24.1	37.9	24.1
STH 5.8436	3	7	10	4	24	12.5	29.2	41.7	16.7
STH 5.8440	1	13	23	6	43	2.3	30.2	53.5	14.0
STH 5.8449	1	1	9	3	14	7.1	7.1	64.3	21.4
STH 5.8450	9	11	7	0	27	33.3	40.7	25.9	0.0
STH 5.8458	5	9	9	4	27	18.5	33.3	33.3	14.8
STH 5.8460	1	2	6	7	16	6.3	12.5	37.5	43.8
∑/Average	50	158	323	169	700	7.1	22.6	46.1	24.1

**Figure 3.**
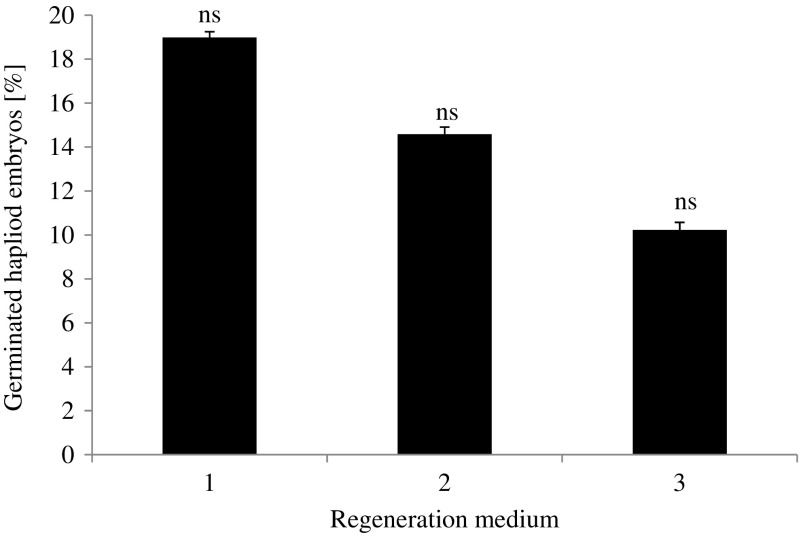
The percentage of germinated oat haploid embryos depending on the regeneration medium. Medium 1: 190–2 with 0.5 mg L^−1^ KIN and 0.5 mg L^−1^ NAA. Medium 2: 190–2 with 1 mg L^−1^ ZEA and 0.5 mg L^−1^ NAA. Medium 3: 190–2 with 1 mg L^−1^ DIC, 1 mg L^−1^ PIC, and 0.5 mg L^−1^ KIN. *ns*, differences not significant according to Duncan test at *p* ≤ 0.05. *Bars* represent SD.

The percentage of germinated haploid embryos of oat differed slightly for each type of regeneration medium, although the differences in the germination of haploid embryos were not statistically significant (Fig. 3). The highest percentage (about 19%) of germinated haploid embryos was observed for those growing on the 190–2 medium containing 0.5 mg L^−1^ KIN and 0.5 mg L^−1^ NAA. The lowest percentage (about 11%) of germinated haploid embryos was observed for the embryos growing on the 190–2 medium containing 1 mg L^−1^ DIC, 1 mg L^−1^ PIC, and 0.5 mg L^−1^ KIN.

**Figure 4.**
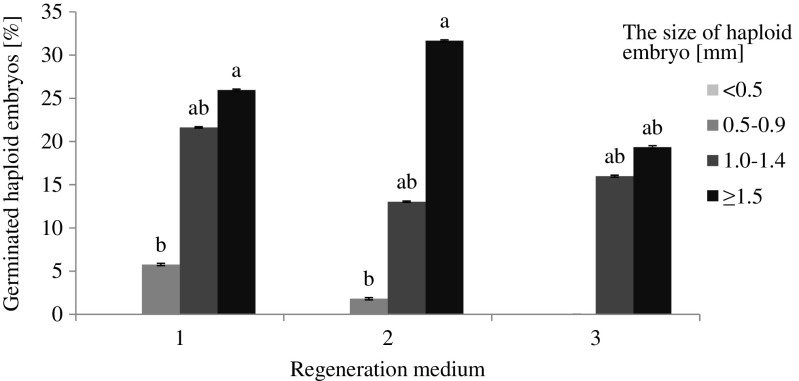
The percentage of germinated oat haploid embryos depending on their size and regeneration medium. Medium 1: 190–2 with 0.5 mg L^−1^ KIN and 0.5 mg L^−1^ NAA. Medium 2: 190–2 with 1 mg L^−1^ ZEA and 0.5 mg L^−1^ NAA. Medium 3: 190–2 with 1 mg L^−1^ DIC, 1 mg L^−1^ PIC, and 0.5 mg L^−1^ KIN. Significant differences between the four groups of embryos according to Duncan test, *p* ≤ 0.05 are marked with different letters. *Bars* represent SD.

Germination capacity varied depending on the size of the embryos (Fig. 4). Larger embryos and those with fully developed coleoptiles and radicles showed a better ability to germinate. The largest germination capacity was exhibited by embryos ≥1.5 mm, whereas those <0.5 mm did not germinate. This relationship was observed for all types of regeneration media. Germination was also not observed for 0.5–0.9-mm embryos transferred to the 190–2 medium containing 1 mg L^−1^ DIC, 1 mg L^−1^ PIC, and 0.5 mg L^−1^ KIN. Comparison of the effect of different regeneration media on embryo germination was performed between the four groups of embryos separately for each medium. The germination capacity of 1.0–1.4-mm and ≥1.5-mm embryos growing on the medium with 0.5 mg L^−1^ KIN and 0.5 mg L^−1^ NAA did not differ significantly from the other two embryo size groups, whereas germination of 0.5–0.9-mm embryos was statistically different from the other groups on that medium. Germination of all embryos transferred to the medium containing 1 mg L^−1^ ZEA and 0.5 mg L^−1^ NAA was significantly different among all size groups. Germination capacity of 1.0–1.4-mm and ≥1.5-mm embryos on the medium containing 1 mg L^−1^ DIC, 1 mg L^−1^ PIC, and 0.5 mg L^−1^ KIN did not differ significantly.

## Discussion

In higher plants, the production of interspecific hybrids depends not only on the ability of parental-species genes to harmoniously cooperate during the development of the embryo, but also their impact on the concerted interaction between the embryo, endosperm, and embryo tissues (Zenkteler 2001). Interspecific crosses frequently lead to poor development of the endosperm or lack thereof (Bridgen 1994) as was observed in this study. Lack of endosperm can cause embryo abortion. This problem may be overcome by aseptic culture of the embryo in nutrient medium which provides nutrients needed for embryo development (Lulsdorf *et al.* 2014). Nutritional requirements depend on the stage of embryo development (Bridgen 1994). Successful production of plants from cultured embryos largely depends on the maturation stage and medium composition (Sharma *et al.* 1996). As far as the stage of their development is concerned, even very immature embryos might be rescued, using complex media. According to Raghavan (1966), two main phases can be identified in the embryo development: the heterotrophic phase (from the zygote to the globular stage of the embryo) and the autotrophic phase (from the cotyledonary stage of the embryo). During the heterotrophic period, the embryo is unable to synthesize nutrition compounds and must receive them from the outside, mainly from the endosperm and the surrounding maternal tissues. During these developmental stages, embryos require more complex media supplemented with a combination of vitamins, amino acids, growth regulators, and, in some cases, natural extracts, such as tomato juice or coconut milk (Bridgen 1994). During the autotrophic phase, the embryo is metabolically capable of synthetizing substances required for its growth from exogenously applied salts and sugars. In this phase, embryos can germinate and grow on a simple inorganic medium supplemented with a carbon source such as sucrose.

The younger the aborting embryo, the more complex are the steps involved in rescuing the embryo and in the medium requirements (Lulsdorf *et al.* 2014). Since removal of young, fragile embryos frequently leads to physical damage; ovule or ovary cultures might be the preferred methods until the embryo reaches more mature stages which is generally past the critical cotyledonary stage. The results of the current study show that although oat haploid embryo isolation was performed at the same time, 21 d after pollination by maize, the embryos were in various developmental stages. Similar results were obtained by Wędzony (1999) with respect to wheat and triticale haploid embryos obtained by pollination with maize. The size of isolated haploid wheat and triticale embryos depended on the developmental phases and genotype. Moreover, globular haploid embryos of wheat and triticale were not able to germinate regardless of the genotype and type of regeneration medium; however, obtaining plants from these haploid embryos was possible by the induction and regeneration of the callus derived from them. In a study by Cherkaoui *et al.* (2000), very small (< 0.5 mm) and poorly structured durum wheat haploid embryos did not germinate when placed on medium, whereas embryos approximately 2 mm in length and with a defined structure and vigorous appearance demonstrated the highest germination capacity. It is in agreement with this study, which showed that germination capacity was strongly correlated with the size of oat haploid embryo.

To study the influence of auxin and cytokinin on the onset and initial development of haploid embryos, embryos were transferred into media differing in growth regulators. In general, low concentrations of auxins have favored normal growth whereas their higher concentrations either proved inhibitory or favored unorganized callus growth from the cultured embryos (Sharma *et al.* 1996). Effects of cytokinins have usually resulted in growth inhibition; however, in this study, equal amounts of KIN and NAA or higher amounts of ZEA than NAA stimulated of growth in cultured haploid embryos of oat. According to Fischer and Neuhaus (1996), auxin seems to be necessary for the establishment of a normal embryonic symmetry at the globular and early transition stages. Their results indicate that exogenously added auxins to early transition embryos initiated lateral promeristem rather than the proper apical wheat promeristem development. In the current study, depending on the growth regulators in the media, the embryos with developed coleoptiles and radicles germinated most efficiently on medium with 1 mg L^−1^ ZEA and 0.5 mg L^−1^ NAA, whereas smaller embryos germinated most efficiently on medium with 0.5 mg L^−1^ KIN and 0.5 mg L^−1^ NAA. These results are consistent with the work of Wędzony (1999), who demonstrated the best germination capacity for haploid embryos of wheat and triticale transferred to a medium supplemented with 1 mg L^−1^ ZEA and IAA or with 0.5 mg L^−1^ NAA and 0.5 mg L^−1^ KIN. Although growth regulators, such as auxins and cytokinins, are extensively used, their effects have been found to be quite inconsistent and at times, contradictory. Since the effects of these substances are not nutritional, it is likely that osmotic concentration and the action of these substances are linked in some way with cell permeability and uptake of ions (Sharma *et al.* 1996).

## Conclusions

The efficiency of oat haploid embryo germination in this study depended on the developmental stage of the embryos and growth regulators added to the regeneration medium. However, further modification of growth regulator contents in the media is required to stimulate the development of globular embryos and to improve the conversion of haploid embryos (with visible coleoptiles and radicles).

## Electronic supplementary material

Table S1 (DOCX 18 kb)

Table S2 (DOCX 16 kb)
