# Supplementary material for: Conversion of oat (Avena sativa L.) haploid embryos into plants in relation to embryo developmental stage and regeneration media
Source: In Vitro Cell Dev Biol Plant. 2016 Nov 4;52(6):590–7. doi: 10.1007/s11627-016-9788-z (PMC5159443; doi:10.1007/s11627-016-9788-z)
Supplement: Supplementary file 1 — (DOCX 18 kb) [file 11627_2016_9788_MOESM1_ESM.docx]

**Table S1**. The number of plants, emasculated florets, isolated haploid embryos, germinated haploid embryos and haploid plants growing on MS0 medium, in perlite and in the soil, DH plants after colchicine treatment, and the obtained oat DH lines

| Genotype | The number of | | | | | | | | |
| --- | --- | --- | --- | --- | --- | --- | --- | --- | --- |
|  | Plants | Emasculated florets | Isolated embryos | Germinated embryos | Haploid plants MS0 | Haploid plants in perlite | Haploid plants in soil | DH plants after colchicine treatment | DH lines |
| STH 4.8456/1 | 51 | 1019 | 29 | 3 | 2 | 2 | 2 | 2 | 2 |
| STH 4.8456/2 | 45 | 1043 | 27 | 10 | 10 | 7 | 7 | 6 | 6 |
| STH 4.8457/1 | 42 | 887 | 30 | 1 | 1 | 1 | 1 | 1 | 1 |
| STH 4.8457/2 | 47 | 1141 | 47 | 8 | 8 | 5 | 4 | 4 | 4 |
| STH 5.8421 | 35 | 998 | 32 | 8 | 8 | 5 | 5 | 5 | 5 |
| STH 5.8422 | 15 | 487 | 5 | 2 | 2 | 0 | 0 | 0 | 0 |
| STH 5.8423 | 39 | 1014 | 47 | 8 | 8 | 3 | 3 | 3 | 2 |
| STH 5.8424 | 30 | 874 | 44 | 6 | 6 | 2 | 2 | 2 | 0 |
| STH 5.8425 | 40 | 1284 | 83 | 21 | 21 | 10 | 9 | 7 | 5 |
| STH 5.8426 | 26 | 738 | 33 | 5 | 5 | 3 | 2 | 1 | 1 |
| STH 5.8427 | 31 | 1145 | 46 | 11 | 11 | 6 | 6 | 5 | 5 |
| STH 5.8428 | 35 | 690 | 11 | 2 | 2 | 1 | 1 | 0 | 0 |
| STH 5.8429 | 34 | 1253 | 64 | 22 | 20 | 10 | 7 | 6 | 6 |
| STH 5.8430 | 17 | 307 | 17 | 3 | 3 | 1 | 1 | 1 | 1 |
| STH 5.8432 | 32 | 698 | 28 | 4 | 4 | 2 | 1 | 1 | 1 |
| STH 5.8436 | 39 | 799 | 24 | 3 | 3 | 2 | 2 | 1 | 1 |
| STH 5.8440 | 35 | 817 | 42 | 7 | 7 | 2 | 2 | 1 | 1 |
| STH 5.8449 | 38 | 844 | 21 | 4 | 4 | 3 | 2 | 2 | 2 |
| STH 5.8450 | 30 | 628 | 27 | 0 | 0 | 0 | 0 | 0 | 0 |
| STH 5.8458 | 34 | 727 | 27 | 4 | 4 | 3 | 2 | 2 | 2 |
| STH 5.8460 | 25 | 511 | 16 | 1 | 1 | 0 | 0 | 0 | 0 |
| ∑ | 720 | 17904 | 700 | 133 | 130 | 68 | 59 | 50 | 45 |
