# Supplementary material for: Conversion of oat (Avena sativa L.) haploid embryos into plants in relation to embryo developmental stage and regeneration media
Source: In Vitro Cell Dev Biol Plant. 2016 Nov 4;52(6):590–7. doi: 10.1007/s11627-016-9788-z (PMC5159443; doi:10.1007/s11627-016-9788-z)
Supplement: Supplementary file 2 — (DOCX 16 kb) [file 11627_2016_9788_MOESM2_ESM.docx]

**Table S2.** The percentage of isolated haploid embryos, germinated haploid embryos, haploid plants on MS0 medium, in perlite and in the soil, DH plants after colchicine treatment, and the obtained oat DH lines per emasculated floret

| Genotype | The percentage of | | | | | | |
| --- | --- | --- | --- | --- | --- | --- | --- |
|  | Embryos/floret | Germinated embryos/floret | Haploid plants on MS0/floret | Haploid plants in perlite/floret | Haploid plants in soil/floret | DH plants after colchicine treatment/floret | DH lines/floret |
| STH 4.8456/1 | 2.85 | 0.29 | 0.20 | 0.20 | 0.20 | 0.20 | 0.20 |
| STH 4.8456/2 | 2.59 | 0.96 | 0.96 | 0.67 | 0.67 | 0.58 | 0.58 |
| STH 4.8457/1 | 3.38 | 0.11 | 0.11 | 0.11 | 0.11 | 0.11 | 0.11 |
| STH 4.8457/2 | 4.12 | 0.70 | 0.70 | 0.44 | 0.35 | 0.35 | 0.35 |
| STH 5.8421 | 3.21 | 0.80 | 0.80 | 0.50 | 0.50 | 0.50 | 0.50 |
| STH 5.8422 | 1.03 | 0.41 | 0.41 | 0.00 | 0.00 | 0.00 | 0.00 |
| STH 5.8423 | 4.64 | 0.79 | 0.79 | 0.30 | 0.30 | 0.30 | 0.20 |
| STH 5.8424 | 5.03 | 0.69 | 0.69 | 0.23 | 0.23 | 0.23 | 0.00 |
| STH 5.8425 | 6.54 | 1.64 | 1.64 | 0.78 | 0.70 | 0.55 | 0.39 |
| STH 5.8426 | 4.47 | 0.68 | 0.68 | 0.41 | 0.27 | 0.14 | 0.14 |
| STH 5.8427 | 4.02 | 0.96 | 0.96 | 0.52 | 0.52 | 0.44 | 0.44 |
| STH 5.8428 | 1.59 | 0.29 | 0.29 | 0.14 | 0.14 | 0.00 | 0.00 |
| STH 5.8429 | 5.11 | 1.76 | 1.60 | 0.80 | 0.56 | 0.48 | 0.48 |
| STH 5.8430 | 5.54 | 0.98 | 0.98 | 0.33 | 0.33 | 0.33 | 0.33 |
| STH 5.8432 | 4.15 | 0.57 | 0.57 | 0.29 | 0.14 | 0.14 | 0.14 |
| STH 5.8436 | 3.00 | 0.38 | 0.38 | 0.25 | 0.25 | 0.13 | 0.13 |
| STH 5.8440 | 5.26 | 0.86 | 0.86 | 0.24 | 0.24 | 0.24 | 0.24 |
| STH 5.8449 | 2.49 | 0.47 | 0.47 | 0.36 | 0.24 | 0.24 | 0.24 |
| STH 5.8450 | 4.30 | 0.00 | 0.00 | 0.00 | 0.00 | 0.00 | 0.00 |
| STH 5.8458 | 3.71 | 0.55 | 0.55 | 0.41 | 0.28 | 0.28 | 0.28 |
| STH 5.8460 | 3.13 | 0.20 | 0.20 | 0.00 | 0.00 | 0.00 | 0.00 |
| Average | 3.82 | 0.67 | 0.66 | 0.33 | 0.29 | 0.24 | 0.22 |
